# Hysterectomy Profile in King Edward Memorial Hospital, Pune, India: Indications, Routes of Surgery, and Complications

**DOI:** 10.7759/cureus.52031

**Published:** 2024-01-10

**Authors:** Shrutika S Khapre, Vivek Joshi, Mangesh D Hivre

**Affiliations:** 1 Department of Obstetrics and Gynecology, Jawaharlal Nehru Medical College, Datta Meghe Institute of Higher Education and Research, Wardha, IND; 2 Department of Obstetrics and Gynecology, King Edward Memorial Hospital, Pune, IND; 3 Department of Surgery, Mahatma Gandhi Institute of Medical Sciences, Wardha, IND

**Keywords:** paralytic ileus, laparoscopic hysterectomy, vaginal hysterectomy, abdominal hysterectomy, fibroids, hysterectomy

## Abstract

Introduction

Hysterectomy is the most common procedure performed in females worldwide in response to a variety of indications. Abdominal and vaginal hysterectomies are the most common routes preferred but laparoscopic hysterectomy is one of the minimal access methods that are being used more often for hysterectomies. Additionally, there are numerous postoperative complications associated with hysterectomies; therefore, the goal of the present study was to determine the indications, commonly preferred routes of surgery, and associated postoperative complications in hysterectomy.

Methodology

A prospective observational study was carried out for 14 months in 2018 and 2019. Based on the selection criteria 120 patients who underwent hysterectomy were recruited for the study in which indications for hysterectomy, route of surgery, and associated postoperative complications were assessed.

Results

The age range of 36-45 accounted for the greatest number of hysterectomies consisting of 47 patients (39.2%) out of 120 participated. Hypertension was the most commonly associated comorbidity in 33 patients (27.5%). The most common indication for hysterectomy was a fibroid in 34 (28.3%) patients and the most preferred route of surgery was through the abdomen in 52 (43.3%) patients. The postoperative complications were more in peripartum hysterectomy and least in vaginal hysterectomy.

Conclusion

Although hysterectomy is frequently performed to enhance quality of life, it can also be a life-saving treatment. As there is a chance of problems with any surgical operation, the indication needs to be carefully considered. Since there are now a lot of conservative methods available for treating benign gynecological disorders, it is wise to talk to the patient about her options before deciding to remove her uterus surgically.

## Introduction

Globally, hysterectomy is one of the most common major surgical procedures performed on female patients [1]. There are no national statistics on hysterectomy rates in India, although prior research indicates that a survey carried out in the northern state of Haryana revealed a 7% prevalence of hysterectomy among married women over the age of 15 years [2]. An additional study conducted in the western state of Gujarat revealed that, at an average age of 37 years, 7% of women in rural areas and 5% of women in urban areas already had a hysterectomy [3].

In India, women prioritize their families before their personal health. The need to have the desired family size often postpones the decision to have one's uterus removed, even in cases where the patient has a uterine condition. In India, therefore, even when a hysterectomy is indicated and recommended, surgery is typically postponed until the children are old enough and the mothers feel satisfied with their familial responsibilities. Age-related increases in the frequency of hysterectomy peaked in the range between 45 and 54 years of age [4-7].

Uterine leiomyomas are the most common reason for hysterectomies, followed by fibroids, menorrhagia, pelvic pain, and uterine prolapse. Adenomyosis, endometriosis, premalignant uterine or cervical disease, and chronic pain are the other relatively prominent indications. Vaginal or abdominal routes have been used for hysterectomy procedures conventionally. Laparoscopic hysterectomy is one of the minimal access methods that are being used more often for hysterectomies [8,9].

Hysterectomy carries the same risks as other surgeries in terms of both intraoperative and postoperative consequences. Hysterectomy-related complications have been documented at rates ranging from 0.5% to 43% [10]. Following a hysterectomy, an estimated 13-37% of women reported that their sexual lives deteriorated [11]. Dyspareunia may arise from a shortened vaginal vault following a hysterectomy, especially if the closure is horizontal (from anterior to posterior). On the other hand, vaginal dryness and decreased libido may be brought on by an estrogen and testosterone shortage resulting from a hysterectomy with bilateral oophorectomy [12,13]. It has been proposed that internal orgasms are inhibited when the cervix is removed (complete hysterectomy). Compared to abdominal hysterectomy, vaginal hysterectomy has been linked to higher sexual morbidity. Even in cases where the ovaries are preserved, the mean age at which menopause begins is 3.7 years earlier in patients who have had hysterectomy [14-16].

Thus, the study's objective was to examine the indications, postoperative complications, and correlation between the preoperative diagnosis and the final histopathological report of all the hysterectomies carried out at King Edward Memorial Hospital, Pune, India. The current study may serve as a foundation for future research in gynecological practice and for comparing our practice with other studies, as there are no recent hysterectomy studies published from India.

## Materials and methods

A prospective observational study was carried out at King Edward Memorial Hospital, Pune, India, between 2018 and 2019. The study included patients who had undergone total abdominal hysterectomy (TAH) involving hysterectomy with unilateral salpingo-oophorectomy (TAH with USO) or bilateral salpingo-ovariotomy/oophorectomy (TAH with BSO), vaginal hysterectomy with or without pelvic floor repair, non-descent vaginal hysterectomy (NDVH) for indications other than uterovaginal prolapse, and a group that underwent laparoscopic hysterectomy that included both total laparoscopic hysterectomy (TLH) and laparoscopic-assisted vaginal hysterectomy (LAVH). Additionally, patients who underwent radical hysterectomy, hysterectomy done as a part of staging laparotomy for ovarian tumor, and peripartum hysterectomy were included in the study. The exclusion criteria involved suspicion of uterine sarcoma, other pelvic organ malignancies, sacro-uterine nodularities, endometriosis, tubo-ovarian abscesses, pregnancy, uterine immobility in the pelvic examination, uterine size above the umbilicus, and history of colorectal surgery.

The total hysterectomies performed were 210 but based on the inclusion and exclusion criteria a total of 120 patients were included in the present study. Patient characteristics like age, parity, body mass index, and presence of co-morbidities were documented, as the indication for which hysterectomy was resorted to, investigations done for the workup of the case involving routine as well as specific investigations, whether medical treatment was given before hysterectomy for relief of symptoms, the details of medical treatment like the drug used and the duration of medical treatment were also documented. Indication for surgery, approach, and whether the approach was influenced by factors like uterine and adnexal pathology, previous abdominal surgery, previous vaginal deliveries, type of anesthesia given, type of incision in abdominal hysterectomy, and the incidence of surgical site infection and associated morbidity and factors influencing the same were recorded. Additionally, other surgical interventions like Burch colposuspension done concomitantly with hysterectomy, whether ovaries were removed or not in accordance with the indication for hysterectomy, operative time, duration of antibiotic use, duration of catheterization, intra-operative and early postoperative complications, and length of hospital stay was also documented. Furthermore, intensive care admissions and repeatedly performed laparotomies were also assessed.

The data on continuous variables is demonstrated as mean and standard deviation whereas, the data on categorical variables is illustrated as n (% of cases). Fisher's exact probability test for a 2 x 2 contingency table or the Chi-square test is used for the inter-group comparison of categorical variables. The analysis of variance (ANOVA) technique is used to determine the statistical significance of the inter-group variation in the means of continuous variables. The underlying assumption of normality was examined prior to putting the research variables via an ANOVA. To enhance the visual representation of the statistically significant difference, the findings are presented in tabular and graphical formats. P-values less than or equal to 0.05 were considered statistically significant. For every null hypothesis (the hypothesis of no difference), two-tailed alternatives were used to generate the hypotheses. The IBM SPSS Statistics for Windows, Version 21 (Released 2012; IBM Corp., Armonk, New York, United States) for Microsoft Windows is used to statistically evaluate all of the data.

## Results

Among the 120 women included in the study, the highest number of hysterectomies were performed for the women in the age group between 36 and 45 years consisting of 47 (39.2%) hysterectomies. The complete set of cases under study had a mean age of 46.9 ± 10.9 years, with a minimum and maximum age range of 24 to 80 years. Parity distribution of cases undergoing hysterectomy during the study period revealed a maximum number of patients with parity two.

Distribution of associated comorbidity among the cases undergoing hysterectomy reported that a maximum number of cases were of hypertension 33 (27.5%), followed by lower segment cesarean section 27 (22.5%), diabetes 26 (21.7%), thyroid dysfunction 12 (10%), anemia 10 (8.3%), HIV 5 (4.2%), tuberculosis and heart disease with 3 (2.5%) each, bronchial asthma 2 (1.6%), and 1 (0.8%) each for syphilis, bronchitis, Crohn’s disease, deep venous thrombosis, dengue, hepatitis B, hepatitis E, and herpes zoster. The distribution of indications for hysterectomy among the cases undergoing hysterectomy is demonstrated in Table 1.

**Table 1:** Indications for hysterectomy

Indications	Number of patients (n)	Percentage (%)
Fibroid	34	28.3
Dysfunctional uterine bleeding	18	15
Prolapse	17	14.2
Adenomyosis	10	8.3
Endometriosis	1	0.8
Ovarian neoplasm (benign and malignant)	7	5.8
Endometrial carcinoma	8	6.7
Cervical intraepithelial neoplasia	1	0.8
Cervical cancer	2	1.7
Postmenopausal bleeding	7	5.8
Chronic pelvic inflammatory disease	1	0.8
Endocervical fibroid polyp	3	2.5
Post-partum hemorrhage	3	2.5
Placenta accreta	3	2.5
Placenta percreta	5	4.2
Total	120	100.00

Out of total 120 patients recruited for the study only 45 patients (37.5%) received medical treatment before hysterectomy that mainly involved progesterone pills 13 (28.9%), followed by preoperative blood transfusion 10 (22.2%), oral contraceptive pills 9 (20.0%), tranexamic acid 6 (13.3%), preoperative antibiotics 3 (6.7%), vaginal packing with estrogen cream 2 (4.4%), estrogen pills 1 (2.2%), and testosterone 1 (2.2%). The distribution of women as per the route of surgery is demonstrated in Table 2.

**Table 2:** Routes of surgery for hysterectomy

Route of surgery	Number of cases (n)	Percentage (%)
Abdominal		
Abdominal hysterectomy	46	38.3
Abdominal+burch colposuspension	5	4.2
Abdominal (extrafascial)	1	0.8
Total	52	43.3
Vaginal		
Vaginal hysterectomy	8	6.7
Vaginal+colpoperineorraphy	9	7.5
Non-descent vaginal hysterectomy	30	25.0
Total	47	39.2
Laparoscopic		
Total laparoscopic hysterectomy	2	1.7
Laparoscopy-assisted vaginal hysterectomy	5	4.2
Total	7	5.9
Radical hysterectomy	3	2.5
Peripartum hysterectomy	11	9.2
Total	14	11.7

The distribution of the route of hysterectomy according to the indication for hysterectomy is demonstrated in Table 3.

**Table 3:** Distribution of route of hysterectomy according to indication for hysterectomy VH+PFR: Vaginal hysterectomy with pelvic floor repair; NDVH: Non-descent vaginal hysterectomy; LAVH: Laparoscopic-assisted vaginal hysterectomy; TLH: Total laparoscopic hysterectomy; DUB: Dysfunctional uterine bleeding; CIN: Cervical intraepithelial neoplasia; PID: Pelvic inflammatory disease; PPH: Post-partum hemorrhage

	Abdominal hysterectomy	Staging laparotomy	Radical hysterectomy	VH+PFR	NDVH	LAVH	TLH
Fibroid (n=34)	21 (61.8%)	-	-	-	11 (32.4%)	-	2 (5.9%)
Prolapse (n=17)	-	-	-	17 (100%)	-	-	0
DUB (n=18)	6 (33.3%)	-	-	-	9 (50%)	3 (16.7%)	0
Adenomyosis (n=10)	5 (50%)	-	-	-	5 (50%)	-	0
Endometriosis (n=1)	1 (100%)	-	-	-	-	-	0
Endocervical fibroid polyp (n=3)	1 (33.3%)	-	-	-	2 (66.7%)	-	-
Ovarian neoplasm (benign and malignant) (n=7)	5 (71.4%)	2 (28.6%)	-	-	-	-	-
Endometrial cancer (n=8)	-	7 (87.5)	1 (12.5)	-	-	-	0
CIN (n=1)	1 (100%)	-	-	-	-	-	0
Cervical cancer (n=2)	-	-	2 (100%)	-	-	-	0
Postmenopausal bleeding (n=7)	3 (42.9%)	-	-	-	2 (28.6%)	2 (28.6%)	0
PID (n=1)	-	-	-	-	1 (100%)	-	0
PPH	3 (100%)	-	-	-	-	-	0
Placenta accreta	3 (100%)	-	-	-	-	-	-
Placenta percreta	5 (100%)	-	-	-	-	-	-
Total (n =120)	54	9	3	17	30	5	2

The distribution of type of incision in the abdominal approach for hysterectomy is demonstrated in Table 4.

**Table 4:** Distribution of type of incision in abdominal approach for hysterectomy

Type of incision	Abdominal hysterectomy		Abdominal hysterectomy+Burch colposuspension		Extra-fascial hysterectomy		Radical hysterectomy		Peripartum hysterectomy		Total
	No. of cases	% of cases	No. of cases	% of cases	No. of cases	% of cases	No. of cases	% of cases	No. of cases	% of cases	
Pfannenstiel incision	22	47.8	-	-	-	-	-	-	-	-	22
Cherney’s incision	5	10.9	5	100	-	-	3	100	-	-	13
Paramedian incision	5	10.9	-	-	1	100	-	-	11	100	17
Midline vertical Incision	14	30.4	-	-	-	-	-	-	-	-	14
Total	46	100	5	100	1	100	3	100	11	100	66

**Figure 1:**
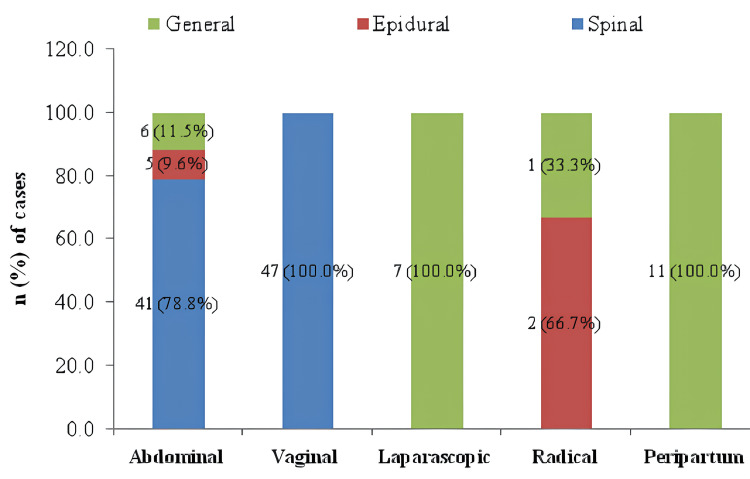
Distribution of patients according to the types of anesthesia used in various routes of hysterectomy

The distribution of 120 patients according to the type of anesthesia used in various routes of hysterectomy is illustrated in Figure 1.

The distribution of mean duration of surgery through various routes of hysterectomy described that mean duration was more in radical hysterectomy (160 mins), followed by peripartum (158.2 mins), abdominal (131.5 mins), laparoscopic (128.6 mins), and vaginal (97.7 mins). Additionally, the distribution of mean duration of hospital stay in various routes of hysterectomy demonstrated that the mean duration of hospital stay was more in peripartum hysterectomy (14.1 days), followed by radical (10.7 days), abdominal (8.1 days), vaginal (6 days), and laparoscopic (5.3 days). Additionally, the distribution of mean duration of use of antibiotics in various routes of hysterectomy reported peripartum hysterectomy with more number of days (15.5 days), followed by radical (11.7 days), abdominal (8.2 days), laparoscopic (7.4 days), and vaginal (7.0 days). Furthermore, the distribution of mean duration of catheterization in various routes of hysterectomy was more in peripartum hysterectomy (16.6 hours), followed by radical (7.7 hours), abdominal (3.3 hours), vaginal (2.4 hours), and laparoscopic (2.1 hours). The distribution of postoperative complications according to the route of hysterectomy is described in Table 5.

**Table 5:** Distribution of postoperative complications according to the route of hysterectomy ICU: Intensive care unit; *p-value<0.05; ***p-value<0.001; NS: Statistically non-significant

	Route of hysterectomy										
	Abdominal (n=52)		Vaginal (n=47)		Laparoscopic (n=7)		Radical (n=3)		Peripartum (n=11)		p-value
Complications	n	%	n	%	n	%	n	%	N	%	
Fever after 24 hours	1	1.9	0	0.0	0	0.0	0	0.0	0	0.0	0.592^NS^
Paralytic ileus	5	9.6	0	0.0	0	0.0	1	33.3	0	0.0	0.066^NS^
Blood transfusions	2	3.8	2	4.2	0	0.0	0	0.0	8	72.7	0.001^***^
Suture line infection	4	7.7	0	0.0	0	0.0	0	0.0	0	0.0	0.116^NS^
Bladder injury	0	0.0	0	0.0	0	0.0	0	0.0	2	18.2	0.999^NS^
Bowel injury	0	0.0	0	0.0	0	0.0	1	33.3	0	0.0	0.999^NS^
Urinary tract infection	2	3.8	0	0.0	0	0.0	0	0.0	0	0.0	0.314^NS^
Respiratory infection	3	5.8	0	0.0	0	0.0	0	0.0	1	9.1	0.201^NS^
Thrombophlebitis	2	3.8	1	2.1	0	0.0	0	0.0	0	0.0	0.785^NS^
Intraoperative blood loss	1	1.9	1	2.1	0	0.0	0	0.0	0	0.0	0.928^NS^
Pelvic hematoma	0	0.0	1	2.1	0	0.0	0	0.0	0	0.0	0.531^NS^
ICU admission	0	0.0	1	2.1	0	0.0	0	0.0	0	0.0	0.531^NS^
Total	20	38.5	6	12.8	0	0	2	66.7	8	72.7	

The correlation between clinical and histopathological findings is demonstrated in Table 6.

**Table 6:** Correlation between clinical and histopathological findings HPE: Histopathological examination

Indication	Histopathological report		HPE in the remaining cases
	Confirmed no. of cases	%	
Adenomyosis (n=10)	5	50	
Fibroid (n=34)	26	76.5	8-adenomyosis
Endocervical fibroid polyp (n=3)	3	100	
Endometriosis (n=1)	1	100	
Carcinoma endometrium (n=8)	8	100	
Benign ovarian pathology (n=5)	3	60	1-left adnexal mass was a subserosal uterine fibroid 1-tubo-ovarian abscess, there was bilateral salphingoophoritis
Ovarian malignancy (n=2)	1	50	1-tuberculosis
Cervical carcinoma in situ (n=1)	1	100	
Carcinoma cervix (n=2)	2	100	
Morbidly adherent placenta (n=8)	7	87.5	1-retained placental tissue
Pelvic inflammatory disease (n=1)	0	0	1-endometrial polyp
Total (n=75)	57	76	n=18

## Discussion

The goal of the current prospective observational study was to determine the route of hysterectomy, indication of hysterectomy, and postoperative complications of hysterectomy in 120 patients who were recruited for the present study. In the present study, the majority of patients who underwent hysterectomy were in the age group between 36 and 45 years which was congruous with the study given by Radha et al. in which the incidence of hysterectomy was maximum in the age group of 36-45 years and the incidence declined with advancing age [17]. In contrast to the current study, Sinhasane and Gm observed a rise in the proportion of hysterectomies with advancing age, with the age group of over 46 years accounting for the largest number of patients who underwent hysterectomy [18].

In the current study, the majority of patients had abdominal hysterectomy, which was followed by vaginal, peripartum, laparoscopic, and radical hysterectomy. This was comparable to earlier research by Pandey et al., where the majority of hysterectomies were performed via the abdominal route, followed by the vaginal and laparoscopic routes [19]. Almost the same observations were reported in previous studies in which the abdominal route was more commonly preferred, followed by vaginal and laparoscopic [18,20].

The most frequent indication for a hysterectomy, according to a comparison of indications with earlier research, was fibroids, which was consistent with research from Pandey et al. [19]. Similarly, studies performed in the United States, Hong Kong, Pakistan, Africa, and Canada also report fibroids as the most common indication for abdominal hysterectomy along with genital prolapsed, uterovaginal prolapsed, and dysfunctional uterine bleeding (DUB) for vaginal hysterectomy [18,20-24].

When medicinal therapy is not tolerated or does not work for a patient, a hysterectomy is preferably considered an option for treatment. Some patients may benefit from alternatives to hysterectomy, such as endometrial ablation or resection, which are less expensive and less likely to cause complications. About 29% of patients assigned to the ablation group received a hysterectomy within 60 months after the start of a clinical trial that randomized endometrial ablation to hysterectomy [25].

Similarly, when comparison of route of surgery in relation to indication for hysterectomy was observed in the study given by Pandey et al., hysterectomies for fibroid uterus were predominantly performed by the abdominal route, followed by LAVH, NDVH, vaginal, and laparoscopic hysterectomies which is in contrast with the present study that demonstrated most common as abdominal route, followed by NDVH, TLH reporting a shift in trend towards less invasive mode of surgery. Similarly, in the present study maximum number of hysterectomies for DUB was through the NDVH route, followed by the abdominal route, and through laparoscopically whereas, in a study by Pandey et al. in which maximum hysterectomies were performed abdominally, followed by LAVH, and NDVH. In the present study, adenomyotic uteri were removed by the abdominal route, followed by NDVH. Whereas, in the study given by Pandey et al., the majority were through the abdominal route, followed by laparoscopically and NDVH [19].

In the present study after abdominal hysterectomy the most common complications observed were febrile morbidity and paralytic ileus, postoperative blood transfusion, surgical site infections, urinary and respiratory tract infections, thrombophlebitis, and excessive intraoperative blood loss. In radical and peripartum hysterectomies bowel and bladder injury, paralytic ileus, and lower respiratory tract infections were commonly observed. Whereas, in a study given by Pandey et al., bowel and bladder injury, ureteric injury, wound infection or gaping, burst abdomen, pelvic abscess, postoperative intestinal obstruction, and blood loss were more commonly observed and required multiple transfusions in abdominal hysterectomy [19].

The incidence of complications was much lower after vaginal hysterectomy which involved excessive intraoperative blood loss, pelvic hematoma, thrombophlebitis, and anesthesia-related complications. Maternal mortality was seen in peripartum hysterectomy. In both cases, the cause of death was not related to the obstetric indication or the complications of peripartum hysterectomy. According to Peipert et al., febrile morbidity is more common after abdominal hysterectomy than it is after vaginal or laparoscopic procedures [26]. After abdominal surgery, one of the most frequent and anticipated complications is postoperative paralytic ileus. Patients who have a vaginal hysterectomy regain normal bowel function more quickly than those who have an abdominal hysterectomy. The duration of hospital stay is significantly influenced by the existence of a postoperative ileus [27]. Bowel motility can also be impacted by inflammatory mediators that are generated in response to surgical manipulation and infections. The standard postoperative treatment regimen includes nasogastric (NG) tube decompression and bowel rest [28].

As NDVH has minimal morbidity and does not require sophisticated equipment, the feasibility of NDVH should be considered in all women requiring hysterectomy for benign uterine diseases. After NDVH, if there is any suspicion of hemorrhage, the introduction of a laparoscope is mandatory not only to control hemorrhage but also to confirm the integrity of ureters. In placenta percreta, the morbidity due to hemorrhage and urological injuries can be diminished by doing a deferred hysterectomy six weeks after classical cesarean section.

The two categories of management options are supportive care and prevention. One can change the surgical approach, the anesthetic being used, and the method of pain treatment in order to prevent it. Much research has examined alternatives to regular NG intubation for supportive care, including prokinetic drugs, early oral feeding, and early ambulation [29]. Proper exposure of the surgical field, dissection in proper tissue planes, and securing surgical knots are some of the methods to decrease intraoperative blood loss. Intraoperative blood loss can be reduced by performing a hysterectomy in the postmenstrual period, and by techniques like hydro dissection in vaginal hysterectomy to facilitate dissection through the proper tissue planes. If bleeding occurs during surgery, the bleeding vessel should be located and carefully ligated, if required with ureter visualization.

In the present study, the mean operative time was shortest in vaginal hysterectomy and longest in abdominal hysterectomy. Similar findings were noted in the study by Tanveer et al. in 2016 [30]. A study by Billfeldt et al. in 2018 noted shortest median operative time in vaginal hysterectomy and longest median operative time in laparoscopic hysterectomy [31]. Studies by Uccella et al. in 2018 [32] and Uccella et al. in 2016 [33] comparing the median operative time between abdominal and laparoscopic hysterectomy have noted that in comparison to abdominal hysterectomy, laparoscopic hysterectomy is linked to a longer median operational time. All of the aforementioned research came to the conclusion that vaginal hysterectomy takes less time to perform than the laparoscopic approach.

In the current study, patients who had an abdominal hysterectomy had the longest mean hospital stay, while patients in whom a laparoscopic hysterectomy was performed reported the shortest mean hospital stay. The studies by Pandey et al. [19] and Toma et al. [21] revealed similar results along with Tanveer et al. in 2016 [30]. Research by Uccella et al. in 2016 [33] and Uccella et al. in 2018 [32] compared the median length of hospital stay following laparoscopic and abdominal hysterectomies and found that laparoscopy through the abdominal route is associated with a longer median length of hospital stay than the laparoscopic route. In conclusion, compared to vaginal hysterectomy, laparoscopic hysterectomy is linked to a shorter hospital stay. Moreover, the present study is associated with certain limitations, which involve a small sample size as the study was performed in only one setting; therefore, multiple settings consisting of various hospitals in the region can be considered for future studies. Another limitation involves the absence of research on long-term complications like bowel and bladder functions, quality of life, sexual life, pelvic pain, and prolapse after hysterectomy for which it is therefore recommended to perform larger studies for research on long-term complications of different hysterectomy procedures.

## Conclusions

The present study reported that the majority of patients were in the age range of 36 to 45 years old. The most commonly associated co-morbidity that was noted was hypertension, while fibroid was the most prevalent indication for a hysterectomy. As a form of medical treatment before hysterectomy majority of the patients received progesterone pills. Abdominal surgery was the most frequently performed surgical approach, followed by vaginal and laparoscopic procedures. Additionally, in the most common indication of hysterectomy that involved fibroid uterus the preferred route was abdominal and the most common incision made was Pfannenstiel with spinal anesthesia. The mean duration of surgery was longer in radical hysterectomy and least in vaginal which was also associated with the least duration of antibiotic use. The postoperative complications were more in peripartum hysterectomy and least in vaginal hysterectomy. Additionally, peripartum hysterectomy required a longer hospital stay and catheterization than laparoscopic hysterectomy. The histopathological examination of the specimen revealed that the majority of cases had the same pathology as suspected preoperatively. Preoperative investigations missed the diagnosis of endometrial malignancy in some of the women who presented with postmenopausal bleeding. Therefore, these investigations including endometrial sampling should not be completely relied upon in cases of postmenopausal bleeding, wherein suspicion of malignancy is high.
